# Link between Quality of Life and Self-Esteem in Tunisian Women with Systemic Lupus Erythematosus

**DOI:** 10.1192/j.eurpsy.2025.1564

**Published:** 2025-08-26

**Authors:** N. Mseddi, R. Ben Jmaa, O. Bouattour, I. Châari, L. Aribi, F. Charfeddine, F. Frikha, J. Aloulou

**Affiliations:** 1 Psychiatry B; 2 Internal Medecine, Hédi Chaker Hospital, Sfax, Tunisia

## Abstract

**Introduction:**

Systemic lupus erythematosus (SLE) is a chronic, multisystemic disease that can significantly impact the quality of life (QoL) and self-esteem of lupus patients.

**Objectives:**

To study the quality of life of female patients with SLE and its relationship with self-esteem.

**Methods:**

A descriptive and analytical cross-sectional study was conducted on 50 female patients with SLE followed at the Department of Internal Medicine at Hédi Chaker Hospital, Sfax, Tunisia, from September to November 2023. We used:

- A socio-demographic and clinical data sheet and the Lupus QoL questionnaire to assess the quality of life of lupus patients
- The Rosenberg Self-Esteem Scale

**Results:**

- The mean age of patients was 43.46 ± 11 years. 56% had a medical or surgical history.
- The mean age of SLE diagnosis was 33.54 years with a mean number of flares of 2.94 years ± 2.473
- **According to the Lupus QoL questionnaire:** the mean global score was 59.76± 23.496 [8.8-91.98]. The mean scores in the different domains were: physical health = 58.35 ± 32.571 [0-97], pain = 53.013 ± 33.896 [0-100], planning= 69.33 ± 32.741 [0-100], feeling of being a burden = 71.36 ± 32.694 [0-100], emotional health = 57.71 ± 26.67 [0-95.83], body image = 72.9 ± 24.6 [10-100], fatigue = 40.93 ± 24.462 [0-87.5].
- **According to the Rosenberg Scale:** a mean global score of 28.28 ± 8.572 [2-40]. Very low to low self-esteem was found in 50% of patients.
- Statistically significant correlations between low scores in the domains of quality of life and low to very low self-esteem were found:
  - Physical health: P = 0.000
  - Planning: P = 0.042
  - Pain: P = 0.002
  - Emotional health: P = 0.004
  - Fatigue: P = 0.017
  - Body image: P = 0.007
  - Global Lupus QoL: P = 0.001

**Conclusions:**

Our results indicate that SLE can impair the QoL of lupus patients in various domains. The same is true for self-esteem. The positive relationship between the two parameters encourages a better understanding of the factors associated with each and highlights the importance of a global approach to improve the management of female lupus patients.

**Disclosure of Interest:**

None Declared

